# Application of a New Alloy and Post Processing Procedures for Laser Cladding Repairs on Hypereutectoid Rail Components

**DOI:** 10.3390/ma15155447

**Published:** 2022-08-08

**Authors:** Olivia Kendall, Panahsadat Fasihi, Ralph Abrahams, Anna Paradowska, Mark Reid, Quan Lai, Cong Qiu, Peter Mutton, Mehdi Soodi, Wenyi Yan

**Affiliations:** 1 Department of Mechanical and Aerospace Engineering, Monash University, Melbourne, VIC 3800, Australia; 2 Australian Nuclear Science and Technology Organisation, Sydney, NSW 2234, Australia; 3 School of Civil Engineering, The University of Sydney, Sydney, NSW 2006, Australia; 4 Institute of Railway Technology, Monash University, Melbourne, VIC 3800, Australia; 5 LaserBond Ltd., Melbourne, VIC 3018, Australia

**Keywords:** laser cladding, repair, residual stress, neutron diffraction, hypereutectoid rail

## Abstract

The development of a laser cladding repair strategy is critical for the continued growth of heavy-haul railway networks. Premium hypereutectoid rails have undergone laser cladding using a new martensitic stainless-steel alloy, 415SS, developed for high carbon rails after standard cladding metals were found to be incompatible. Non-destructive neutron diffraction techniques were used to measure the residual stress in different layers generated across a dissimilar metal joint during laser cladding. The internal stress distribution across the cladding, heat-affected zone (HAZ), and substrate was measured in the untempered rail, after 350 °C and 540 °C heat treatment procedures and two surface grinding operations. The martensitic 415SS depositions produce compressive stress in the cladding, regardless of tempering procedures, which may inhibit fatigue crack propagation whilst grinding operations locally relive surface stress. Balancing tensile stresses were recorded below the fusion boundary in the HAZ due to thermal gradients altering the microstructure. The combination of 540 °C tempering and 0.5 mm surface layer removal produced a desirable combination of compression in the cladding deposition with significantly reduced tensile stresses in the HAZ. A comparison with the current literature shows that this alloy achieves a unique combination of desirable hardness, low tensile stress, and compression in the cladding layer. Data obtained during strain scanning has been used to determine the location of microstructural changes at the fusion boundary and HAZ through correlation of the stress, strain, full width at half maximum (FWHM), and intensity profiles. Therefore, neutron diffraction can be used for both the accurate measurement of internal residual stress and to obtain microstructural information of a metallurgical join non-destructively.

## 1. Introduction

Heavy-haul railway lines are continuously subjected to high axle loads at increasing speeds, which accelerates the onset of rolling contact fatigue (RCF) and wear. If rail deterioration is left unaddressed, severe consequences may result in the form of catastrophic failures. Ongoing and mandatory rail maintenance, repair and replacement procedures are both disruptive and costly; therefore, maintenance strategies must be continuously improved to keep railway networks operational. A traditional approach to rail maintenance is grinding which is used to remove damaged surface layers containing defects and potential crack initiation sites [1]. This offers a temporary improvement in rail performance and hence must be repeated at regular intervals; however, over time, this will gradually reduce the rail profile and decrease the rail service life.

Due to its widespread availability and speed, arc welding techniques are commonly employed for overlay welding to rebuild rail components. One less desirable effect of welding is the production of significant heat-affected regions due to high heat inputs [2]. This results in tensile residual stress, softening, and substantial changes to microstructure, all of which increase the susceptibility to cracking during wheel–rail contact [3]. Liu et al. [4] considered the influence of weld-induced residual stress and cyclic loading under wheel–rail contact on the fatigue life of rail joints. Regions of high tensile stress in the rail head and web were found to be more susceptible to cracking whilst compressive zones in the web are beneficial in prolonging the fatigue lifetime. Kabo et al. [5] implemented simulations to assess the influence of weld repairs on the RCF susceptibility. The authors determined highly tensile regions at the base of the weld site and defects at the weld–substrate interface, when combined with high shear stress during wheel–rail contact, may initiate sub-surface RCF. Furthermore, under cyclic contact stress, shallow weld repairs were found by Lee et al. [6] to have a fatigue lifetime ten times lower than deeper repairs due to a 30% increase in residual stress in near-surface rail welds.

As an alternative repair strategy to recondition railway components, laser cladding offers distinct advantages over current welding techniques. Laser cladding is a metal deposition technology which applies a high quality, hard facing layer to the rail surface by metallurgically bonding a metallic powder using a high energy laser. Guo et al. [7] reported a significant decrease in wear-related damage after cladding cobalt-based alloys on wheel and rail steel grades during twin-disc testing. A decreased wear rate was also found by Zhu et al. [8] after cladding stainless steel on wheel sections and subjecting them to twin-disc tribometer testing. Furthermore, Hernández et al. [9] successfully implemented an industrial laser cladding technique in the heat-affected zone (HAZ) of aluminothermic (thermite) welds as a means of extending their service life.

Laser cladding is a highly flexible process that requires a lower heat input compared to standard welding procedures. This produces a small HAZ below the fusion boundary, which decreases microstructural softening and residual stresses. Nevertheless, as cladding is a thermal technique, generation of residual stress is unavoidable. Residual stresses arise due to thermal gradients, localised melting, phase transformations, volumetric changes, solidification shrinkage, and mismatch of the thermal expansion coefficient between the cladding and substrate [10,11]. Processing parameters greatly influence the stress distribution, and thereby tempering procedures may be implemented to relieve internal stress and control the microstructure. Chen et al. [12] used high-temperature heat treatments to alter the microstructure and transition residual stresses from tensile to compressive in laser clad tool steel. Head hardening was also shown by Turan et al. [13] to have a similar effect on R260 rails as increasing the cooling rate increased the magnitude of compressive stresses.

In railway applications, it is the combination of residual and wheel–rail contact stresses that govern rail performance; therefore, low residual and tensile stresses are desirable to reduce the likelihood of fatigue. This was demonstrated by Ringsberg et al. [14] who conducted extensive simulations to assess the influence of residual stress after cladding and grinding operations on RCF behaviour of Co-Cr deposits. The authors concluded that the stress state before twin-disc testing decreases the fatigue life and results in a tensile state in Co-Cr deposits. Roy et al. [15,16] undertook an extensive investigation of the residual stress and wear properties of laser clad premium rail components. High tensile stresses within the Stellite 6 deposition severely decreased the wear resistance due to extensive cracking in the cladding layer, whereas stainless steel depositions contained compressive surface stresses that are more resistant to fatigue crack propagation. Narayanan et al. [17] studied the residual stress redistribution of pearlitic rail clad with a martensitic steel and established a steady stress state after 10 fatigue loading cycles. Compressive stresses within the cladding increased the fatigue resistance, indicating laser cladding can produce components with improved wear and fatigue properties.

To achieve superior wear performance, overmatching of the mechanical properties is common where a high wear resistance accompanies a high hardness, modulus, and brittle microstructure when compared to the substrate [18]. It is vital that new alloys for rail applications are produced that closely match the properties of the substrate metal. A mismatch of hardness or the production of high tensile stresses at the repair site will shift the wearing or stress cracking process elsewhere in the system. Therefore, better matching of cladding properties with the substrate through applying new alloys and post processing procedures is what the rail industry needs.

Accurate measurement of the residual stress is critical in the development of thermal repair processes such as laser cladding. There are several approaches to measure residual stress which can be categorised as destructive or non-destructive. Destructive techniques such as sectioning, hole drilling, and the contour method utilise machining to remove material, thereby relieving internal stress which is measured as a change in strain. Non-destructive techniques, including ultrasonic, magnetic, and diffraction methods, record stress in relation to a material parameter such as magnetism or lattice spacing [3]. Neutron diffraction offers unique advantages over more accessible X-ray diffraction techniques due to the increased penetration capability of neutrons which is up to 50 mm in steel compared to 50 µm for X-ray radiation, which limits X-ray diffraction techniques to near surface measurements [19,20]. This enables internal, triaxial strain to be recorded non-destructively, making it a powerful technique for analysis of laser clad components by enabling sub-surface measurements in the cladding, heat affected regions, and substrate [21].

In this study, a post-cladding heat treatment regimen was developed to control the cladding microstructure and hardness of a new martensitic laser cladding alloy (415SS) for hypereutectoid steel. Two grinding operations were also applied to establish the influence of standard finishing procedures on the internal stress state. The residual stresses within the cladding, HAZ, and substrate after laser cladding were non-destructively assessed using the Kowari neutron diffractometer at ANSTO (Australian Nuclear Science and Technology Organisation). The data obtained from neutron measurements can be used to non-destructively determine the location of the fusion boundary and HAZ which was verified by microstructural analysis. The resultant hardness within the cladding and HAZ as well as the maximum residual stresses were compared with other studies to highlight the critical combination of stress and hardness achieved through implementation of this heat treatment and grinding regimen. This will be used to provide critical information to determine the fatigue performance and operation lifetime of rail components after use of a laser cladding maintenance strategy for repair.

## 2. Materials and Methods

### 2.1. Laser Cladding

**Figure 1:**
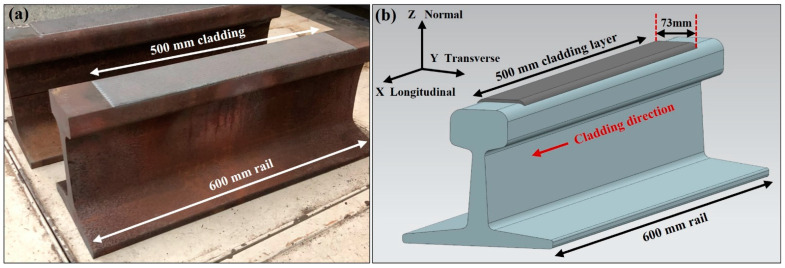
(**a**) Laser-clad hypereutectoid rail used for analysis and (**b**) schematic of a laser-clad rail.

During laser cladding, the metal powder was injected using a Sulzer–Metco twin 10 powder feeder and directed towards the laser beam and rail surface, to form a melt pool before solidifying to create the cladding deposit. Laser cladding of the hypereutectoid steel sections was undertaken using a laser coaxial head and 4 kW IPG fibre laser. Cladding was performed with a 3.2 kW laser power, 5 mm spot size and 1000 mm/s traverse speed. A 50% Argon and 50% Helium shielding gas was used to protect the melt pool from oxidation during the cladding process. These parameters were previously established by Lai et al. [22] for high carbon rail substrates. The 600 mm rail sections were longitudinally laser-clad with a 2.5 mm thick deposition over 500 mm on the 73 mm wide rail head, as shown in Figure 1.

### 2.2. Cladding Materials and Sample Preparation

A new martensitic stainless steel, 415SS, was used as the cladding alloy for the hypereutectoid rail substrates. This cladding material was developed by combining 410L and SS420, as both are promising cladding alloys but were found to be individually incompatible with high carbon rail by Lai et al. [22]. The 410L deposition was reported to produce a ferritic microstructure in the cladding deposition on high carbon rail; therefore, the resultant hardness falls below industry requirements making it unsuitable for this application. In comparison, the SS420 alloy produced significant untempered martensite in the HAZ whilst the cladding hardness was still above industry requirements after heat treatment due to the high carbon composition. Therefore, combining 410L and SS420 was expected to produce a low cost, Fe-based alloy for industrial applications with a moderate hardness to match the substrate material. The composition of 415SS and the substrate rail is presented in Table 1.

Metallographic specimen preparation involved sectioning the rail with an abrasive saw in the transverse direction before mounting, grinding, polishing, and etching with a 2% Nital etchant to expose the microstructure of the rail substrate. Kalling’s no. 2 solution (5 g CuCl_2_, 100 mL HCl and 100 mL ethanol) was used to observe the cladding layer with an Olympus GX optical microscope. The microstructure and residual stress were correlated to the mechanical hardness measured using a Vickers microhardness tester with a 5 kg load.

### 2.3. Heat Treatment Processes

**Figure 2:**
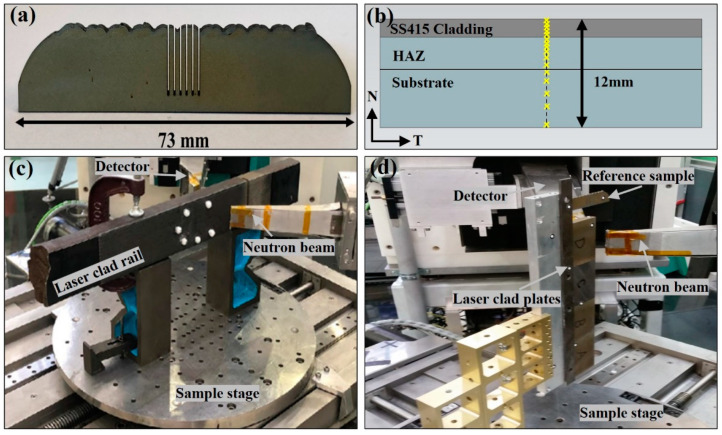
(**a**) The strain free reference sample. (**b**) Strain scanning locations in the transverse rail plane. Longitudinal scanning orientation of the (**c**) 415SS rail and (**d**) plates on the diffractometer stage.

Before cladding, each rail was pre-heated to 350 °C using a manual oxy-fuel torch and monitored with thermocouples to maintain this interpass temperature. This was to prevent rapid cooling during cladding which results in undesirable martensite formation in the HAZ. Previous investigations undertaken at Monash University by Lai et al. [22] applied a low-temperature post-cladding heat treatment by heating the rail to 350 °C after cladding and covering with an insulative blanket to force a slow cooling rate. This was found to reduce the cladding hardness of the SS420 stainless steel depositions, however, was insufficient to produce comparable mechanical properties across the rail. The same heat treatment was applied to the lower carbon 415SS cladding alloy after deposition on the high carbon rail section. Following this tempering process, 60 × 50 × 11 mm^3^ plates were extracted from the rail head using electro-discharge machining (EDM) for residual stress analysis, as shown in Figure 2. This plate size is sufficient to be produced using wire cutting without disrupting the internal stress at the centre where residual stress is to be measured using neutron diffraction. Therefore, the measurements taken in these plates are comparable to those taken on the untempered full-scale rail section. A second high-temperature heat treatment process was developed for the 415SS alloy and implemented on plates after the rail had cooled to room temperature. These plates were held at 540 °C for two hours in a furnace, then left to air cool. The tempering conditions were determined experimentally to achieve an average cladding hardness of 419 HV(5) in accordance with standard tempering parameters applied to martensitic stainless steels. Vickers hardness tests indicated a tempering process was required to achieve compatible mechanical properties with the substrate rail; therefore, grinding was only carried out on tempered rail sections. Two different levels of grinding were applied to the plates after tempering to replicate standard finishing procedures applied to laser clad components for high wearing applications. Grinding was used to remove 0.5 mm and 1.4 mm from the cladding on the plates after the 350 °C and 540 °C tempering processes. The heat treatment and surface grinding conditions of samples used for residual stress measurements are presented in Table 2.

### 2.4. Residual Stress Measurement

Non-destructive residual stress measurements were carried out on the Kowari strain scanner at ANSTO. Double focusing bent crystal Si (400) monochromators were used to select out a single neutron wavelength of 1.67 Å for diffraction. Due to the thickness of the cladding deposition, a gauge volume of 0.5 × 0.5 × 1 mm^3^ was used to ensure this volume would be encompassed within the cladding layer after surface grinding. A lattice plane of Fe-α (211) was used for strain measurements in the cladding and substrate due to similarities of the bulk elastic modulus in iron-based alloys [20].

A strain-free reference sample was prepared using EDM to extract five 0.5 mm thick slices from the rail head and cutting five teeth, 0.5 × 1 × 12 mm^3^ at the centre to relax the internal stresses, as shown in Figure 2a. To reduce the distance between diffracted neurons and the detector, two blind access holes were drilled below the rail head of the full-scale rail section, ending 10 mm below the cladding surface. Roy et al. [16] previously used simulations to show residual stresses at the measurement location are not influenced by machining of blind holes in the rail head which are beneficial in reducing measurement time. To accurately position the gauge volume at each measurement location, the rail was scanned using a coordinate measurement machine to generate a local coordinate system. The samples were then aligned on the diffractometer stage with reference to fiducial markers to map the measurement locations. The sample setup of the full-scale rail and plates are shown in Figure 2c,d.

The grains that are aligned to meet the scattering condition within the gauge volume diffract neutrons that are collected by the detector. The peak scattering angle is identified from the diffraction pattern by Gaussian fitting using QKowari software. This scattering angle is applied to Bragg’s Law shown in Equation (1) below to calculate the lattice spacing $d_{hkl}$, where n is a positive integer and the order of reflection, λ is the neutron wavelength and *θ* is the scattering angle for the crystallographic planes.

(1) $$n\lambda =2d_{hkl}sin\theta$$

An internal tensile or compressive stress generates an expansion or contraction of the lattice spacing with respect to a strain-free reference which causes a relative shift in the diffraction peaks. Determination of the lattice spacing of the reference ($d_{hkl}^{0}$) and at the measurement location ($d_{hkl}$) enables calculation of the strain using the formula:

(2) $$\varepsilon_{hkl}=\frac{d_{hkl}-d_{hkl}^{0}}{d_{hkl}^{0}}$$

The three principal stresses are then calculated using Hooke’s law where *E* = 210 GPa and *υ* = 0.28 for high carbon steel in the following equations,

(3) $$\sigma_{xx}=\frac{E}{\left(1-\upsilon \right)\left(1-2\upsilon \right)}\left[\left(1-\upsilon \right)\varepsilon_{xx}+\upsilon \left(\varepsilon_{yy}+\varepsilon_{zz}\right)\right]$$

(4) $$\sigma_{yy}=\frac{E}{\left(1-\upsilon \right)\left(1-2\upsilon \right)}\left[\left(1-\upsilon \right)\varepsilon_{yy}+\upsilon \left(\varepsilon_{zz}+\varepsilon_{xx}\right)\right]$$

(5) $$\sigma_{zz}=\frac{E}{\left(1-\upsilon \right)\left(1-2\upsilon \right)}\left[\left(1-\upsilon \right)\varepsilon_{zz}+\upsilon \left(\varepsilon_{xx}+\varepsilon_{yy}\right)\right]$$

Through-thickness line scans were performed at the centre of both the rail and plate samples to measure strain in three principal directions ($\varepsilon_{xx},\;\varepsilon_{yy},\;\varepsilon_{zz}$), corresponding to the transverse, normal and longitudinal directions, as shown in Figure 1. Strain was recorded at 16 locations from the component surface and terminated 8 mm below the cladding surface, as shown in Figure 2b.

## 3. Results

### 3.1. Microstructure

**Figure 3:**
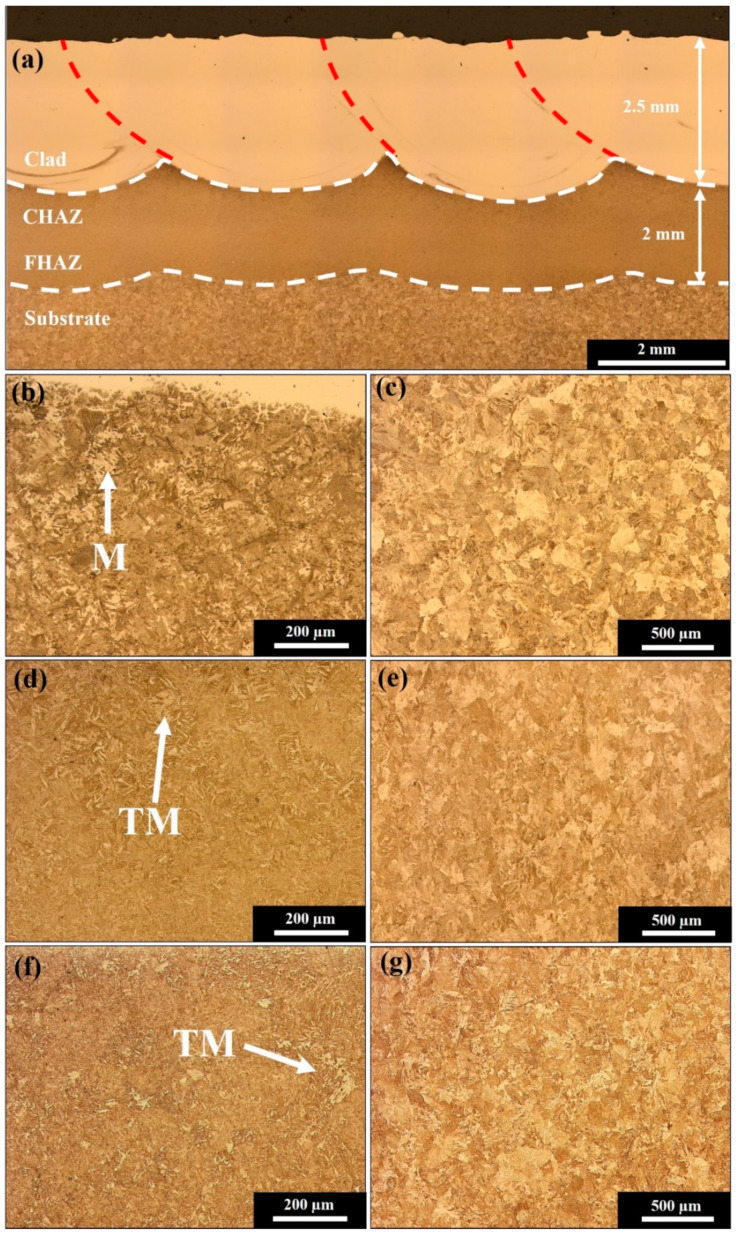
Transverse microstructure showing the (**a**) cladding, coarse (CHAZ), and fine (FHAZ) regions within the HAZ and the rail substrate. The untampered rail contains (**b**) untempered martensite (M) in the CHAZ before reaching the (**c**) pearlitic substrate. The 350 °C heat treatment produces (**d**) tempered martensite (TM) in the HAZ and (**e**) pearlitic substrate. A 540 °C tempering causes a refinement of the (**f**) HAZ without significantly changing the (**g**) pearlitic substrate rail.

The resultant cross-sectional microstructure after laser cladding is presented in Figure 3a. This shows the directions of the laser tracks and distinctive regions of cladding, HAZ, and rail substrate. Due to the thermal input required to melt the substrate rail, large thermal gradients at the surface bring about phase transformations due to the austenisation of the microstructure. A heat-affected region is formed below the cladding-fusion boundary, extending around 2 mm into the parent rail in the untempered state. Below this, the thermal gradient decreases transitioning to the fine-grained HAZ before reaching the pearlitic parent rail substrate.

The untempered rail contains a light phase of martensite in the upper region of the HAZ which coexists with pearlitic microstructure, as indicated in Figure 3b. Gradual cooling of the rail during cladding produces a steep thermal gradient at the rail surface, facilitating rapid cooling sufficient for untempered martensite to form. The amount of martensite decreases further below the cladding interface as this gradient is reduced before reaching the pearlitic substrate shown in Figure 3c.

Tempering at 350 °C and slow cooling have been undertaken to remove HAZ martensite and moderate the cladding properties. The amount of untempered martensite in the coarse region of the HAZ shown in Figure 3d has been reduced, following this low- temperature heat treatment process to produce tempered martensite. Whilst the post-cladding heat treatment was sufficient to remove untempered HAZ martensite, the pearlitic microstructure of the parent rail remains unchanged, as presented in Figure 3e. The 540 °C temperature post-cladding treatment resulted in significant refinement of the HAZ and tempering of the martensite, as shown in Figure 3f. Although the entire plate was exposed to furnace heating, the rail substrate retained the pearlitic microstructure as indicated in Figure 3g, without significant coarsening and has a similar microstructure to the untempered state.

**Figure 4:**
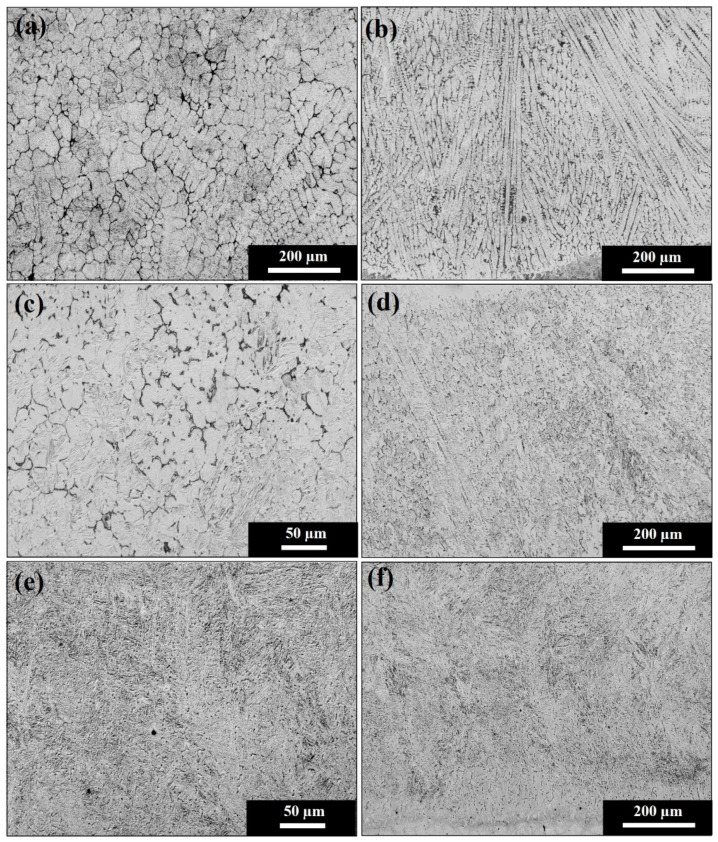
Microstructure of the cladding showing (**a**) equiaxed martensite at the surface of the untempered rail, (**b**) dendritic martensite at the interface. The 350 °C tempered cladding deposition contains (**c**) partially tempered equiaxed martensite at the surface and (**d**) partially tempered dendritic martensite at the interface. The 540 °C heat treatment produces a fully tempered martensitic structure at both the (**e**) surface and (**f**) interface.

Without a post cladding tempering procedure, deposition of the 415SS cladding layer produces a martensitic microstructure, with a cellular morphology at the interface that becomes dendritic before undergoing an equiaxed transition at the surface, as shown in Figure 4a,b. This arises due to changes in the ratio of thermal gradient and velocity of the solidification front. The reduced thermal gradient at the substrate surface forms an equiaxed morphology. In comparison, the higher thermal gradients experienced within the cladding cause directional growth of martensitic grains in the opposite direction to heat flow into the bulk rail, which acts as a heat sink.

Reheating the rail after cladding to 350 °C and slow cooling causes partial tempering throughout the cladding layer. The textured microstructure of the dendritic and equiaxed martensite is partially disrupted due to diffusion brought about by reheating and decreasing the cooling rate to form a more stable martensitic morphology. The result is moderately tempered equiaxed martensite at the cladding surface, as shown in Figure 4c, and partially tempered dendritic martensite towards the cladding interface in Figure 4d. The 540 °C post-cladding heat treatment produced a fully tempered martensitic cladding deposition where the grain boundaries of the previous morphologies at the surface or interface shown in Figure 4e,f can no longer be observed. Holding the plates at this high temperature for two hours allows greater levels of diffusion, enabling a fully tempered morphology to replace the equiaxed and dendritic martensitic structures.

### 3.2. Residual Stress

#### 3.2.1. Residual Stress of the Untempered 415SS Laser Clad Rail

**Figure 5:**
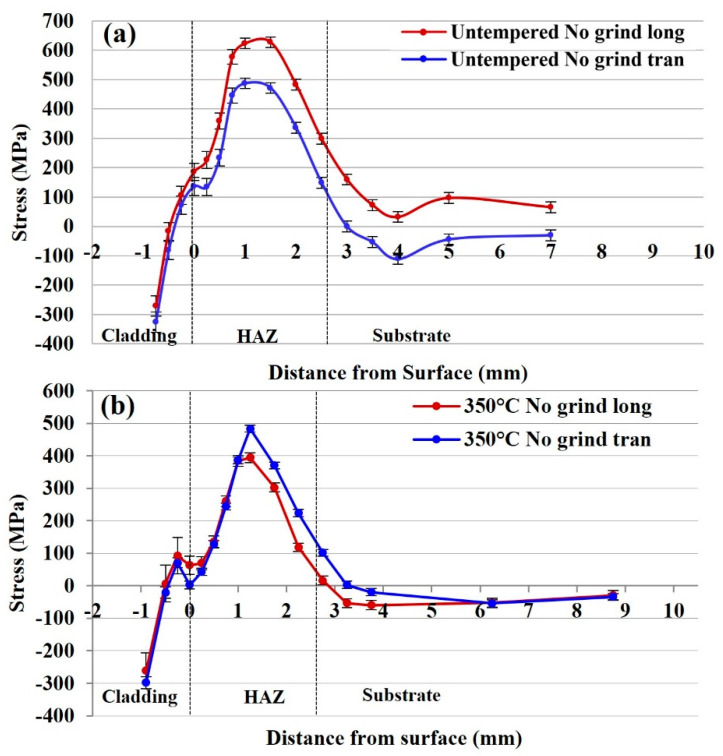
(**a**) Residual stress distribution of the untempered 415SS clad rail and (**b**) residual stress distribution of the 350 °C tempered, 415SS clad rail with no surface grinding. (long—-longitudinal tran—transverse).

To establish the influence of laser clad 415SS on the internal stresses in a high carbon rail, non-destructive strain scanning has been performed using neutron diffraction techniques. Through-thickness line scans were carried out at the centre of the untempered 415SS clad rail, containing two blind access holes to decrease the neutron path length through the sample and increase the output signal. The procedure is described in detail in a previous paper by Roy et al. [16]. Strain measurements from the reference sample indicated retained stresses in the cladding layer after wire cutting. Therefore, the assumption was made that the stress in the vertical direction, normal to the railhead surface was zero and the value of d_0_ that was measured in the normal direction was used to validate the transverse and longitudinal stress measurements close to the surface. Within the substrate, the value from the reference sample was used, however, the normal stress was zero or very close to zero, so it is not shown on the graphs for clarity. The resultant stress profiles for the 600 mm full scale rail section without a post cladding heat treatment are shown in Figure 5a. Transverse and longitudinal measurements indicate the untempered cladding is in a compressive stress state, reaching a maximum of −271 MPa at the surface in the longitudinal direction. This stress increases at the cladding–substrate interface before transitioning to tensile in the HAZ, with a peak value in the longitudinal direction of 627 MPa. This transition occurs due to microstructural changes from heat input at the substrate surface; therefore, the stress begins to neutralise when approaching the unaffected rail 3 mm below the fusion line.

The stress profiles of the 350 °C tempered rail plate with slow cooling are presented in Figure 5b. Compressive stresses were also observed in the cladding deposition with a magnitude of −298 MPa at the surface when compared to the untempered rail at the same location. This stress rapidly decreases when approaching the interface whereupon a maximum tensile stress of 483 MPa is achieved in the transverse direction. The longitudinal stress reaches a peak value of 393 MPa at the same location, 1.25 mm below the cladding–substrate interface. This differs from the stress observed in the rail without a post heat treatment where the greatest stresses occurred in the longitudinal direction. Beyond this heat-affected region, the stress approaches a neutral state at 3.25 mm from the interface.

#### 3.2.2. Residual Stresses of 415SS Laser Clad Rail Plates with 350 °C Tempering and Grinding

**Figure 6:**
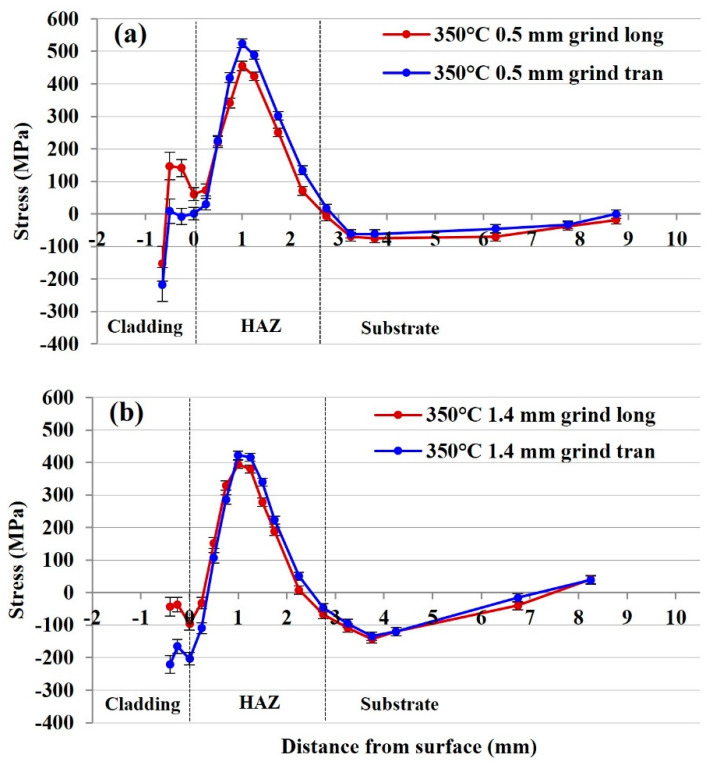
(**a**) Residual stress distribution of the 350 °C tempered, 415SS clad rail with 0.5 mm surface grinding and (**b**) residual stress distribution of the 350 °C tempered, 415SS clad rail with 1.4 mm surface grinding (long—longitudinal tran—transverse).

The longitudinal and transverse stresses from the 350 °C plate with 0.5 mm surface- grinding are shown in Figure 6a. Removal of 0.5 mm from the cladding surface increased the magnitude of the compressive stress in the cladding deposition to −217 MPa in the transverse direction, whilst the maximum longitudinal compressive stress was −153 MPa. A similar rapid transition to tensile stress was observed near the deposition–rail boundary; stress levels then continued to increase within the HAZ until a peak-compensating tensile stress of 524 MPa was reached in the transverse direction, 1.75 mm below the cladding surface. At this site, the longitudinal stress reaches 455 MPa before neutralising 3.25 mm from the fusion line.

Removal of 1.4 mm from the cladding by surface grinding decreased the compressive stresses within the cladding layer to −43 MPa in the transverse and −220 MPa in the longitudinal directions, as shown in Figure 6b. The increased magnitude of the transverse compressive stress coincides with the stress measured in the other 350 °C tempered plates, however, is more pronounced in the samples exposed to grinding. This compressive state remains at the cladding–substrate interface where the transverse stress is −203 MPa before becoming tensile in the heat-affected region. Similar peak tensile stresses of 422 MPa and 394 MPa are obtained in transverse and longitudinal directions. A brief transition to compressive stress of −134 MPa is observed beyond the HAZ, 3.75 mm from the interface before reaching a neutral state. This may occur to compensate the high magnitude of tensile stresses which arise in the HAZ, as the amount of compression in the cladding layer is reduced with greater levels of surface grinding that decreases the thickness of the cladding deposition. In comparison to the untempered rail, transverse stresses appear to dominate the HAZ in rails tempered at 350 °C; however, both conditions show a greater magnitude of transverse compressive stresses at the surface of the 415SS cladding layer.

#### 3.2.3. Residual Stress of the 540 °C 415SS Laser Clad Rail with Surface Grinding

**Figure 7:**
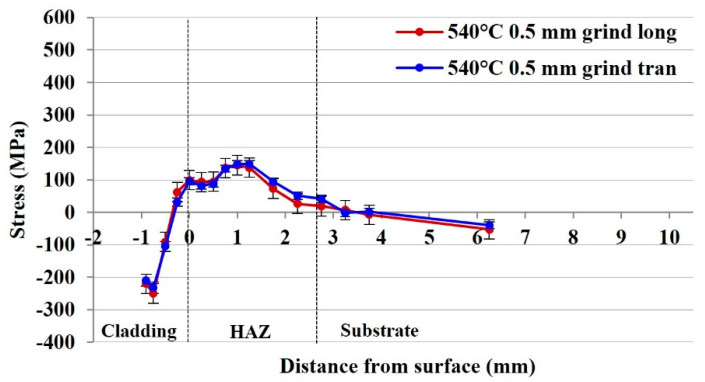
Residual stress distribution of the 540 °C tempered, 415SS clad rail with 0.5 mm surface grinding (log—longitudinal tran—transverse).

Application of a high temperature post-cladding heat treatment results in a significant relief of residual stress in both the cladding and HAZ. Holding the plate at 540 °C for two hours produces a microstructural change in both the cladding and HAZ, facilitating the relief of residual stresses. Further removal of 0.5 mm from the cladding surface produces similar compressive stresses in both the longitudinal and transverse directions of −219 MPa, as shown in Figure 7. These stresses decrease towards the HAZ and retain similar peak tensile values of 148 MPa, 1.25 mm from the deposition–rail interface which is the lowest magnitude of tensile stress achieved in the HAZ under the current tested parameters. The stress continues to decrease and begins to neutralise 3.25 mm from the cladding interface.

### 3.3. Influence of Tempering on Microstructural Hardness

**Figure 8:**
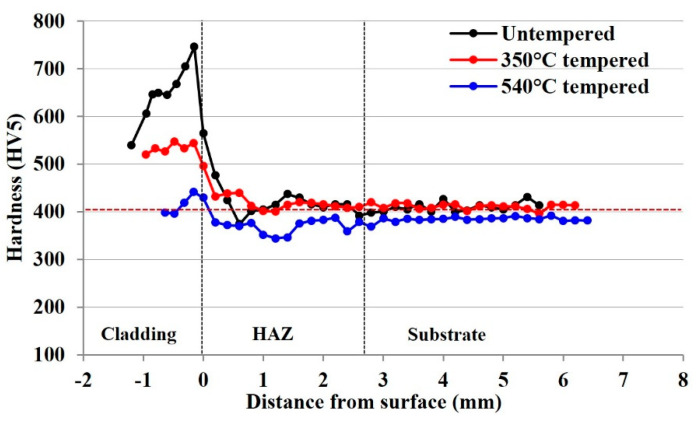
Transverse hardness profiles of the 415SS cladding in the untempered state and after 350 °C and 540 °C heat treatments.

The Vickers hardness profiles of the 415SS laser cladding depositions after high- and low-temperature heat treatment regimens are presented in Figure 8. These profiles have been taken at the centre of the railhead after transverse sectioning which corresponds to the locations of the residual stress measurements. Hardness testing has been performed to correlate the influence of the tempering procedure on the mechanical properties. In the as-clad state, the textured dendritic and equiaxed martensitic microstructure of the cladding deposition results in an average hardness of 653 HV which increases at the cladding fusion boundary due to cellular interfacial martensite. Untempered martensite formation in the coarse-grained HAZ from the gradual cooling of the rail maintains a hardness of 512 HV, before decreasing towards the fine-grained region and stabilising at 400 HV in the pearlitic substrate rail.

Reheating at 350 °C and slow cooling results in partial tempering of the cladding deposition, decreasing the hardness to 520 HV. Microstructural analysis indicates this results in the removal of the untempered martensitic phase in the HAZ, further indicated by a hardness of 450 HV in the coarse-grained region. Approaching the fine-grained HAZ, the hardness is comparable to the untempered state, indicating that this heat treatment process only significantly influences the microstructure and hardness up to 3 mm below the cladding surface. The higher temperature regimen results in a substantial decrease in cladding hardness to 419 HV due to complete tempering of the martensitic 415SS microstructure. A small increase in hardness is observed at the cladding interface, but a similar removal of untempered martensite was achieved in the HAZ, producing an average of 380 HV. A slight reduction in hardness is also observed in the rail substrate, as the plates were exposed to the tempering process; however, no major microstructural changes occurred, and the hardness remains within the specified range for this rail grade.

### 3.4. Identification of Microstructural Changes from Neutron Diffraction Data

Non-destructive neutron diffraction data can be used to obtain microstructural information without the need for destructive analysis or mechanical testing. Examination of the full width at half maximum (FWHM) profiles indicates the broadening of diffraction peaks can be used to obtain additional information from diffraction-based strain measurements using X-ray or neutron energy sources, as was reported by Vashista et al. [23]. Jun et al. [24] found peak-broadening from the FWHM profiles corresponds to regions of rail head plastic deformation and non-uniform strain after repeated rolling contact cycles. Kelleher et al. [25] showed the FWHM and intensity profiles could be correlated to stress or intergranular stress within roller-straightened rails due to plastic deformation generated during manufacturing and operation. The hardness after surface treatment operations on steel has also been related to the FWHM profile by Fu et al. [26].

**Figure 9:**
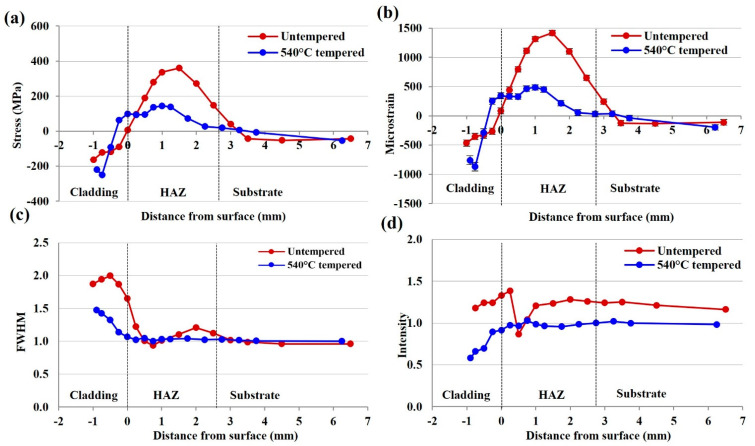
Longitudinal (**a**) stress, (**b**) microstrain, (**c**) FWHM, and (**d**) intensity of the untempered laser clad rail and 540 °C tempered plate.

A comparison of the longitudinal residual stress, strain, FWHM, and intensity profiles for the untempered rail and 540 °C heat-treated plate shown in Figure 9 have been used to determine significant phase changes indicative of a fusion boundary and HAZ. The FWHM profiles contain a significant change in diffraction peak width at the expected location of the cladding–substrate interface, suggesting a microstructural variation which arises from the formation of a dissimilar joint. Without prior knowledge of the cladding thickness, alignment of the FWHM with the macrostrain and intensity captures the locations of the fusion boundary within the rail substrate, indicating the thickness of the cladding deposits before and after surface grinding.

Below this interface lies the HAZ which contains the peak tensile stress. Generally, the FWHM profiles exhibit a greater peak width in regions of high strain at the cladding surface and in the HAZ, around 0.75 mm below the fusion interface. This can arise due to distortion of the crystal lattice and was reported by Filippone et al. [27] to occur due to dislocations and deformation. Tomota et al. [28] also reported changes in peak-broadening with microstructural transformations in steel. The change in FWHM profiles is more prominent in samples with greater microstructural variation such as the as-clad rail. The 540 °C heat treatment reduces tensile stress and refines the HAZ, producing a consistent peak-broadening with the pearlitic substrate and removes significant variation of the FWHM in the HAZ region. However, correlation of this profile with the macrostrain and stress allows the HAZ width in the high-temperature heat-treated sample to be determined. This characterisation of the interfacial positions of the cladding and HAZ after different levels of surface grinding from neutron diffraction data agrees with the locations determined using hardness and microstructural analysis of the cladding, HAZ, and substrate. The ability to correlate the FWHM to the internal stress data indicates the versatility of the neutron diffraction technique to determine residual stress and microstructural changes non-destructively.

## 4. Discussion

### 4.1. Influence of Tempering on the Residual Stress in the Cladding Layer

Neutron diffraction techniques were used for strain measurements in the untempered 415SS laser clad rail to determine the influence of the cladding process on stress generation in the rail head. In the untempered state, compressive stresses were found throughout the cladding which is attributed to the martensitic microstructure of the deposition. The 415SS deposition contains dendritic martensite at the substrate interface which transitions to equiaxed martensite towards the cladding surface where the highest compressive stresses were identified. The formation of martensite is accompanied by the characteristic body-centered tetragonal (BCT) lattice structure, producing a volume expansion that generates these high compressive stresses in the cladding layer [17]. This phenomenon was also observed by Jiang et al. [29] within welding repairs. Moreover, Wang et al. [30] determined that the martensitic transformation alters the residual stress by 200 MPa across a plate structure, and this variation increases depending on the component geometry.

A similar martensitic transformation was reported by Roy et al. [16] to produce compressive surface stresses in 410L clad on premium rail grades. As the rail surface is more vulnerable to damage due to a combination of residual stresses and rolling contact loading, compressive stress may be beneficial in resisting fatigue cracking. Increased energy is required for crack initiation and propagation due to compressive forces acting at a crack tip, thereby increasing the wear and RCF resistance of the rail and prolonging operation life [31].

Microhardness results indicate in the untempered state that the 415SS cladding deposition has a much higher hardness than the rail substrate. A mismatch of mechanical properties is undesirable and may lead to brittle failure of the cladding. The benefits of a post-cladding heat treatment process are threefold, by tempering to moderate the deposition hardness and increase the compatibility with the rail substrate, removing untempered HAZ martensite, and relieving residual stresses. For these reasons, two different tempering procedures were applied to 415SS laser clad rail to moderate the cladding properties.

Reheating the rail to 350 °C with a slow cooling rate results in a compressive stress at the cladding surface of −217 MPa, similar to the untempered state. Tempering facilitates the diffusion of carbon atoms in the supersaturated material lattice to form lower energy phases of cementite and ferrite. This produces a partially tempered martensitic cladding deposition which along with the increased cooling rates at the cladding surface results in compressive stress. Similar findings have been reported by Turan et al. [13] who applied heat treatment processes with varying cooling rates to rail steel, resulting in compressive surface stresses. Alam et al. [32] also implemented a 565 °C post heat treatment to laser clad SS420 steel and determined a shift to higher compressive surface stress compared to the as-clad state with an accompanying decrease in cladding hardness. A higher heat treatment temperature is generally required for martensitic stainless steels to achieve appreciable tempering. This also influences the hardness distribution, as demonstrated by Chen et al. [12] who achieved a tempering-induced hardness decrease in the cladding layer and HAZ after laser cladding Stellite 6 onto tool steel.

After tempering at 540 °C for 2 h, compressive stresses were measured in the cladding deposition of −91 MPa, comparable to those found in the untempered rail. Despite the similarity in residual stress, the microstructure underwent a significant change to achieve a fully tempered martensitic morphology with a corresponding hardness drop to 419 HV that better matches the properties of the substrate rail. Significant stress relief of martensitic stainless steel has been reported to require temperatures between 500–650 °C; therefore, this indicates why a substantial reduction in residual stress state was not observed after the 350 °C post cladding treatment [33].

### 4.2. Influence of Tempering on the Residual Stress State in the HAZ

The compressive state of the cladding layer is accompanied by balancing tensile stresses further within the rail head which may negatively impact the fatigue life by increasing the defect growth rate. This was measured extensively by Kelleher et al. [34], using laboratory and synchrotron X-ray diffraction. These tensile stresses are located within the HAZ below the cladding substrate interface. The HAZ is generated due to localised melting of the substrate surface from the thermal inputs that are sufficient to heat this region above the austenitic transformation temperature, leading to solid state phase transformations upon cooling. Rapid cooling occurs as the underlying bulk rail acts as a heat sink, facilitating the formation to pearlite, bainite, and untempered martensite in the coarse-grained heat-affected region of the untempered rail. Prolonged heating in the HAZ is described by Ishigami et al. [35] to enable phase transformations, softening, and spheroidisation which introduce tension due to thermal and solidification stresses. Volumetric shrinkage and expansion accompanying these microstructural changes has also been extensively discussed by Pandey et al. [36].

As the cladding layer is in a compressive state due to the martensitic transformation and rapid cooling rate at the cladding surface, the softened HAZ becomes tensile to balance this stress. The peak tensile stress of 627 MPa arises 1.5 mm below the fusion boundary in the untempered rail. Similarly, Iyota et al. [37] reported tensile stresses in the HAZ after spot-welding high-strength steel sheets due to a martensitic transformation. Beyond the HAZ, the rail substrate is not exposed to these large thermal gradients and rapid cooling rates which facilitate the formation of martensite; therefore, the magnitude of the tensile stress decreases with increasing pearlite content and the stress state neutralises further away from the HAZ.

The peak tensile stress observed in the HAZ after the 350 °C heat treatment is lower than the as-clad state. Without tempering, cladding of the hypereutectoid rail enables an untempered martensitic phase to form in the HAZ which is known for its high hardness and brittle behaviour. Heat treatment of the cladded rail at 350 °C removes the untempered martensite in this region, significantly decreasing the hardness to 450 HV. This tempering process maintains the compression in the cladding layer due to the volume increase associated with martensite formation and the increased cooling rate at the deposition surface. The softening which occurs due to tempering in the HAZ increases the plasticity in this area, therefore relieves and slightly reduces the tensile stresses required to balance the compressive surface state. The peak tensile HAZ stress occurs in the longitudinal direction in the full-scale rail, however, this peak occurs in the transverse direction in the heat-treated plates. The use of blind access holes to measure stresses within the full-size rail is able to fully constrain the stresses present and so the highest stress is measured in the longitudinal direction, parallel to the length of the rail. In comparison, sectioning of the plates reduces the length of the component in the longitudinal direction and in doing so, slightly relaxes the stresses in that direction too. This results in a higher transverse stress being recorded in the plate samples. As the profiles show the transverse and longitudinal stress components are very similar in both directions, especially close to the surface, this effect is not considered to be significant.

This maximum tensile stress is observed at a similar location, around 2 mm below the cladding surface in both the rail and plate samples. Not only is it desirable for the magnitude of these tensile stress to be low, but it is also preferred for the peak stresses to occur deeper below the rail surface to decrease the susceptibility to RCF-related failures. When cracks do initiate within the cladding layer and propagate through to the fusion boundary, the presence of tensile residual stresses within the HAZ will enhance further crack propagation, and hence, there is a need to minimise these. The peak tensile stresses generated in the HAZ remain below the yield stress of hypereutectoid steel (around 900 MPa [22]) and are therefore not likely to cause fatigue crack propagation under compressive in-service loading. Under in-service rolling contact cycles, the stress state has been reported by Jun et al. [31] to become increasingly compressive and redistribute over the service life, thereby reducing the tensile stress in the railhead. Microstructural analysis of the coarse-grained HAZ suggests the low-temperature heat treatment produces a partial tempering of the HAZ martensite whilst a decrease in mechanical hardness to 450 HV was achieved.

The 540 °C tempering process results in a greater refinement of the HAZ microstructure with complete removal of the untempered martensite. Prolonged tempering allows the HAZ microstructures to transition to more stable phases of cementite which adopts a low energy, spherical morphology in a ferritic matrix. Harisha et al. [38] reported spheroidisation contributes to a relief of thermal residual stresses and increase in ductility, as defects in the crystal lattice are removed. This high-temperature post-cladding heat treatment process resulted in a maximum tensile stress of 148 MPa or a 76% decrease in tensile stress when compared to the as-clad state.

### 4.3. Influence of Surface Grinding on the Residual Stress State in Cladding and HAZ

Surface grinding is a standard finishing procedure for laser cladding repairs on high wearing components such as rails which require a smooth surface finish for wheel contact. Grinding is an abrasive machining process and will result in deformation of the rail surface and produce localised changes in the cladding stress state which may influence the behaviour under wheel–rail contact. Ringsberg et al. [14] implemented laser cladding and grinding simulations on a rail substrate to establish the cladding residual stress transitions to tensile after surface grinding, which was also verified using destructive hole-drilling techniques.

As a heat treatment process is required to achieve mechanical properties that meet industry requirements, the surface grinding procedures were carried out on the plates after tempering. Removal of 0.5 mm of the cladding deposition from the 350 °C tempered plate decreased the magnitude of surface compressive stresses to −217 MPa from −298 MPa in the unground state, corresponding to a 27% decrease in stress. Continued grinding after the 350 °C tempering process to remove 1.4 mm of the cladding further reduces the stress to −165 MPa and achieved a nearly neutral state at the cladding interface. This extensive grinding procedure results in an approximately 40% decrease in stress, however, maintains the desirable state of surface compression using a martensitic stainless-steel cladding alloy.

Grinding is a mechanical procedure and removal of the outer layers of material contributes to the relief of strain. Whilst the magnitude of compressive stress after grinding is reduced in the cladding, this procedure has no effect on the internal stresses below the fusion boundary with the substrate interface. Therefore, it can be surmised that the stress- relieving effect of surface grinding is generally limited to 1 mm below the ground surface. Removal of 0.5 mm from the cladding after the 540 °C heat treatment procedure produced a surface stress of −232 MPa, similar to the other tempering condition and also maintained the compressive state within the cladding. This suggests that a high-temperature heat treatment with surface grinding will result in the best combination of microstructural and mechanical properties whilst producing the lowest stress state. The hardness of the 415SS deposition complements the rail substrate, whilst tempering decreases the peak tensile stresses within the HAZ. The compressive stresses retained in the cladding after surface grinding may impede crack propagation and delay the onset of RCF.

### 4.4. Comparison of Residual Stress and Hardness Results with Other Studies

**Figure 10:**
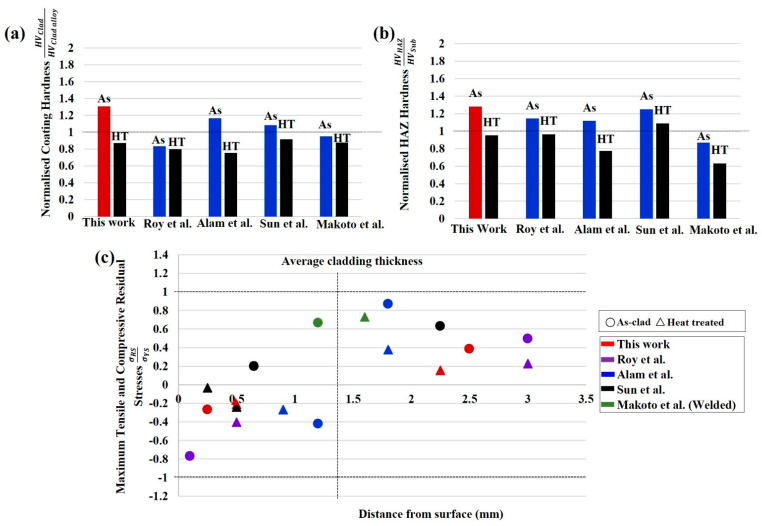
Comparison of 415SS with the current literature showing (**a**) cladding hardness normalised by the hardness of the cladding alloy, (**b**) HAZ hardness normalised by the hardness of the substrate material, (**c**) maximum tensile and compressive stress of laser clad and welded steel normalised by the yield stress of the cladding alloy and substrate material.

After reviewing the literature, a comparison of the findings from this investigation is presented in Figure 10. The average hardness of the coatings and the HAZ has been normalised by the cladding alloy (HV_clad_/HV_clad alloy_) and substrate hardness (HV_HAZ_/HV_sub_) in Figure 10a,b. This indicates the new heat treatment process for 415SS closely matches the parent metal without excessive softening of the cladding or HAZ as the normalised hardness approaches one. The maximum tensile and compressive stress normalised by the yield stress of the cladding and substrate $\left(\sigma_{RS}/\sigma_{YS}\right)$ is also shown in Figure 10c. Laser clad 415SS with the new post weld heat treatment produces low tensile stresses further below the cladding surface when compared to other laser cladding and welding operations [32,39,40]. Additionally, compressive stresses in Figure 10c are located at the cladding surface without producing high tensile stresses within the component, as previously discussed.

Whilst greater compressive stress at the surface has been achieved in other studies, this is at the expense of generating large compensating tensile stresses within the component that may be detrimental to the fatigue lifetime. This suggests the combination of the 415SS alloy with this new tempering process is able to attain a critical combination of cladding and HAZ hardness similar to high carbon steel substrate, compressive surface stresses, and low tensile stresses below the fusion boundary.

## 5. Conclusions

Non-destructive neutron strain scanning has been used to evaluate the effect of two post processing procedures on the microstructure and residual stresses within hypereutectoid rail laser clad with 415SS.

The key findings can be summarised as follows:

- In the untempered state, internal stress within the 415SS deposition is compressive in both the transverse and longitudinal directions due to the martensitic microstructure within the cladding layer. The compressive stress is balanced by tensile stress in the HAZ below the cladding–substrate interface produced by thermal inputs and solid-state phase transformations.
- Tempering at 350 °C after cladding maintains the beneficial compressive surface stresses and generates a higher compensating tensile stress in the transverse direction of the HAZ. Grinding after this heat treatment locally relieves the surface stress, with both 0.5 mm and 1.4 mm grinding depths retaining the compressive stress state in the 415SS depositions.
- A post-cladding heat treatment of reheating and holding at 540 °C for 2 h achieves a significant reduction of 76% in tensile stresses in the HAZ when compared to the untempered rail. Removal of 0.5 mm due to grinding also retains the desirable compressive cladding state which may improve the wear and RCF resistance.
- The location of the fusion boundary and significant microstructural changes after cladding and tempering can be identified through correlation of the stress profiles to the FWHM, strain, and intensity data. Therefore, non-destructive neutron diffraction measurements can be used to obtain both residual stress and microstructural information from a metal joint.

A comparison of laser clad 415SS on high carbon steel with the literature highlights the significant advantages of the alloy combination with the high-temperature post processing regimen for railway applications. This suggests 415SS is a viable cladding alloy for high carbon steel after high-temperature heat treatment and surface grinding operations and can achieve desirable microstructure and hardness with compressive cladding stresses and +reduced tensile stress in the HAZ. These findings will be used to validate finite element models to allow a more effective way to assess cladding quality for safe railway operation and develop post processing procedures for new cladding applications.

## Figures and Tables

**Table 1:** Chemical composition of the 415SS cladding and hypereutectoid substrate in weight percent (wt%).

Alloy	Fe	Cr	C	Mn	Si	Ni	Cu	Mo	V	Nb	Al	S	P	Ti
415SS	Bal	12.4	0.13	0.85	0.48	0.21	0.04	0.03	0.01	<0.01	<0.01	0.01	0.02	<0.01
Rail	Bal	0.20	0.93	0.95	0.28	<0.01	-	<0.01	<0.01	<0.01	<0.01	0.014	-	-

Alloy

Fe

Cr

C

Mn

Si

Ni

Cu

Mo

V

Nb

Al

S

P

Ti

**Table 2:** Tempering and surface grinding conditions of the samples for residual stress measurement.

Sample	Cladding	Tempering Process	Surface Grinding
Rail (600 mm section with 400 mm cladding)	415SS single layer	untempered	No grinding
Plate (60 × 50 × 11 mm^3^)	415SS single layer	350 °C and slow cooling	No grinding
Plate (60 × 50 × 11 mm^3^)	415SS single layer	350 °C and slow cooling	0.5 mm removed
Plate (60 × 50 × 11 mm^3^)	415SS single layer	350 °C and slow cooling	1.4 mm removed
Plate (60 × 50 × 11 mm^3^)	415SS single layer	540 °C for 2 h, air cooling	0.5 mm removed

## Data Availability

Not applicable.
